# Dr. Colburn's New Base for Artificial Teeth

**Published:** 1867-10

**Authors:** 


*Dr. Colburn's New Base for Artificial Teeth.*—This material, of which a notice was given in the September No. of the Journal, is of the nature of a cement—being composed mostly of mineral substances, and containing none of the elements of Rubber or Gutta Percha. Asbestos appears to be an important ingredient, and it is said to possess "remarkable toughness, adherence, strength and lightness." The specimen we have seen, a full upper set of teeth, was composed of this cement attached to an aluminum plate, and presented a very fine appearance. Of its durability we know nothing, but Dr. Colburn in his circular refers to the certificates of responsible persons who have satisfactorily worn for several months, sets of teeth mounted upon this base. He also asserts that "single teeth or blocks can readily be mounted upon a plate of aluminum by means of this material, as they could be attached to gold or platina." "The teeth are arranged as for Rubber work, the plate struck up, its surface well roughened and placed in the flask; no backing or soldering being required." It is also asserted that the fluids of the mouth have had no effect upon it, and that in some mouths, the polish instead of being removed by wearing, is increased.

We present the above statement to our readers in order that they may be able to form some idea of this new material, and what is claimed for it.

